# K27M mutation in histone H3.3 defines clinically and biologically distinct subgroups of pediatric diffuse intrinsic pontine gliomas

**DOI:** 10.1007/s00401-012-0998-0

**Published:** 2012-06-03

**Authors:** Dong-Anh Khuong-Quang, Pawel Buczkowicz, Patricia Rakopoulos, Xiao-Yang Liu, Adam M. Fontebasso, Eric Bouffet, Ute Bartels, Steffen Albrecht, Jeremy Schwartzentruber, Louis Letourneau, Mathieu Bourgey, Guillaume Bourque, Alexandre Montpetit, Genevieve Bourret, Pierre Lepage, Adam Fleming, Peter Lichter, Marcel Kool, Andreas von Deimling, Dominik Sturm, Andrey Korshunov, Damien Faury, David T. Jones, Jacek Majewski, Stefan M. Pfister, Nada Jabado, Cynthia Hawkins

**Affiliations:** 1 Department of Human Genetics, McGill University, Montreal, QC Canada; 2 The Arthur and Sonia Labatt Brain Tumour Research Centre, The Hospital for Sick Children, 555 University Avenue, Toronto, ON M5G 1X8 Canada; 3 Division of Experimental Medicine, McGill University, Montreal, Canada; 4 Division of Haematology–Oncology, The Hospital for Sick Children, Toronto, Canada; 5 Department of Laboratory Medicine and Pathobiology, Faculty of Medicine, University of Toronto, Toronto, Canada; 6 McGill University and Genome Quebec Innovation Center, Montreal, Canada; 7 Department of Paediatrics, Montreal Children’s Hospital, McGill University Health Center, Montreal, QC H1P 2P3 Canada; 8 Department of Pathology, Montreal Children’s Hospital, McGill University Health Center, Montreal, QC H1P 2P3 Canada; 9 Division of Molecular Genetics, The German Cancer Research Center (DKFZ), Heidelberg, Germany; 10 Division of Pediatric Neuro-oncology, The German Cancer Research Center (DKFZ), Heidelberg, Germany; 11 Division of Pathology, The Hospital for Sick Children, Toronto, Canada; 12 Clinical Cooperation Unit Neuropathology, The German Cancer Research Center (DKFZ), Heidelberg, Germany; 13 Department of Hematology and Oncology, Heidelberg University Hospital, Heidelberg, Germany

**Keywords:** DIPG, H3.3, ATRX, TP53, Survival, Targeted therapy

## Abstract

Pediatric glioblastomas (GBM) including diffuse intrinsic pontine gliomas (DIPG) are devastating brain tumors with no effective therapy. Here, we investigated clinical and biological impacts of histone H3.3 mutations. Forty-two DIPGs were tested for H3.3 mutations. Wild-type versus mutated (K27M-H3.3) subgroups were compared for *HIST1H3B*, *IDH*, *ATRX* and *TP53* mutations, copy number alterations and clinical outcome. K27M-H3.3 occurred in 71 %, *TP53* mutations in 77 % and *ATRX* mutations in 9 % of DIPGs. *ATRX* mutations were more frequent in older children (*p* < 0.0001). No G34V/R-H3.3, IDH1/2 or H3.1 mutations were identified. K27M-H3.3 DIPGs showed specific copy number changes, including all gains/amplifications of *PDGFRA* and *MYC/PVT1* loci. Notably, all long-term survivors were H3.3 wild type and this group of patients had better overall survival. K27M-H3.3 mutation defines clinically and biologically distinct subgroups and is prevalent in DIPG, which will impact future therapeutic trial design. K27M- and G34V-H3.3 have location-based incidence (brainstem/cortex) and potentially play distinct roles in pediatric GBM pathogenesis. K27M-H3.3 is universally associated with short survival in DIPG, while patients wild-type for H3.3 show improved survival. Based on prognostic and therapeutic implications, our findings argue for H3.3-mutation testing at diagnosis, which should be rapidly integrated into the clinical decision-making algorithm, particularly in atypical DIPG.

**Electronic supplementary material:**

The online version of this article (doi:10.1007/s00401-012-0998-0) contains supplementary material, which is available to authorized users.

## Introduction

High-grade astrocytomas [anaplastic astrocytoma and glioblastoma (GBM)] are the most biologically aggressive form of cancer and a leading cause of cancer-related mortality and/or morbidity in the pediatric years [15, 19, 22]. They account for 20 % of all brain tumors in children and occur mainly supratentorially in the cortex or thalamus or in the brainstem where they are called diffuse intrinsic pontine gliomas (DIPGs). Cortical GBM can be amenable to complete surgical resection, however, up to 85 % will die within 2 years of diagnosis [3, 15, 21]. DIPGs cannot be surgically removed, because of their location and the infiltrative nature of the disease. They have a median survival of <1 year, with fewer than 10 % of children surviving for more than 2 years [9, 13]. In addition, based on their infiltrative nature and location within the brainstem, DIPGs are often diagnosed clinically based on a combination of neurological signs, duration of symptoms and specific neuro-imaging findings. Currently, biopsy of these tumors is controversial as the findings do not alter therapy if the child presents with classic clinical and imaging features. However, biopsy may be helpful if biological information gleaned from the tissue may guide therapy or provide additional prognostic information.

Despite aggressive therapeutic approaches and decades of clinical trials evaluating numerous chemotherapeutic and radiation therapy regimens, there has been no improvement in survival for children with GBM. The impediment to treatment is the invasive capacity of these high-grade astrocytomas within the brain and their inherent resistance to adjuvant therapies. In addition, permanent damage inflicted to a developing brain by current life-saving therapies severely impacts the quality of life of surviving children [4, 8, 10].

A number of recent comprehensive studies have reported differences at both the copy number and expression levels that distinguish pediatric DIPG from both their adult and pediatric supratentorial GBM counterparts, indicating that they may be separate biologic entities [1, 17, 26]. These studies also identified frequent up-regulation of receptor tyrosine kinases (RTKs) in DIPGs, in particular PDGFR-alpha, MET and IGF1R. These RTKs are also over-expressed in supratentorial GBM, albeit at much lower levels. These findings spearheaded several ongoing clinical trials targeting these RTKs, however, initial results show similarly the poor response rates to those seen to previous, more standard therapies [11]. This suggests that RTK-inhibition alone may not be sufficient to combat DIPG.

We [23] and others [25] recently identified mutations in histone H3.3 (gene name *H3F3A*) at either amino acid 27, resulting in replacement of lysine by methionine (K27M), or at amino acid 34, resulting in replacement of glycine by valine or arginine (G34V/R), as molecular drivers of a subgroup of pediatric and young adult GBM. We also showed in supratentorial pediatric GBM that H3.3 mutations significantly overlapped with mutations in *TP53* and *ATRX* (alpha-thalassemia/mental-retardation syndrome-X-linked) [23], which encodes a subunit of a chromatin remodelling complex required for H3.3 incorporation at pericentric heterochromatin and telomeres [7, 12]. K27M mutations in H3.3, or in the related H3.1, were additionally found in 60 and 18 % of DIPGs, respectively [25]. Here, we investigate the frequency of these mutations in a large series of 42 DIPGs. We additionally assess whether *ATRX* mutations are prevalent in DIPG, and whether they overlap with histone H3.3 and/or *TP53* mutations similar to our findings in supratentorial GBM [23]. Lastly, we investigate the clinical and biological features of DIPG subgroups based on histone H3.3 mutation status.

## Patients and methods

### Patients and samples

**Fig. 1:**
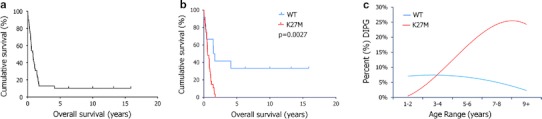
K27M-H3.3 is associated with worse overall survival and higher age of diagnosis in DIPG. **a** Kaplan–Meier curve of overall survival for all DIPG patients (*n* = 39). **b** DIPG patients carrying K27M-H3.3 mutation have worse overall survival compared to patients wild-type for this histone as determined by Kaplan–Meier analysis (Log-rank, *p* = 0.0027). Notably, all long term survivors were wild-type for *H3F3A*. **c** Age distribution of DIPG patients based on K27M-H3.3 mutational status. DIPG patients mutated for K27M-H3.3 have a higher age of diagnosis 8.13 years (±3.75) as compared to wild-type patients [4.57 years (±4.07), *p* = 0.010]

Patient biological material was collected from the Hospital for Sick Children in Toronto, Canada, The Montreal Children’s Hospital/McGill University Health Center in Montreal, Canada, and from the German Cancer Research Center (DFKZ) in Heidelberg, Germany. The study was approved by the Institutional Review Boards of the respective hospitals. Patients were included if they had classic DIPG MRI findings and clinical presentation, including short duration of symptoms (classic DIPG), or had atypical MRI findings and/or clinical presentation (atypical DIPG) but had biopsies demonstrating high grade astrocytoma. Cases were independently reviewed by senior pediatric neuropathologists (CH, SA, AVD) according to the WHO guidelines. Sixteen of the DIPG samples were pre-treatment biopsies, 25 were post-treatment autopsy specimens and one sample was collected at autopsy from an untreated patient (DIPG02). The mean age of diagnosis was 7.12 years (range 0–17 years) with a median survival of 0.83 years (Fig. 1a). Clinical characteristics of patients are summarized in Table 1. All patients were considered and treated as DIPGs in their respective centres. Forty patients had astrocytomas (38 high-grade and 2 grade II). The other two cases had no immunohistochemical evidence of glial differentiation and were labeled as primitive neuroectodermal tumors based on autopsy. Clinical characteristics of the 48 pediatric supratentorial GBMs were previously described [23].

**Table 1:** Patient characteristics and mutational status of samples for H3.1, H3.3, *ATRX*, *TP53* and *IDH1/2*

Sample ID	Age Dx	Gender	OS (years)	Path Dx	Treatment	H3.1	H3.3	*ATRX*	*TP53*	*IDH1/2*	SNP array
DIPG04	10.6	M	0.85	GBM	Post	WT	K27M	MUT	MUT	WT	SNP6.0
DIPG05	8.9	F	1.00	GBM	Post	WT	K27M	–	MUT	–	SNP6.0
DIPG06	7.2	M	0.50	GBM	Post	WT	K27M	WT	MUT	WT	SNP6.0
DIPG07	5.0	M	0.55	GBM	Post	WT	K27M	WT	MUT	WT	SNP6.0
DIPG08	5.8	F	0.44	GBM	Post	WT	K27M	WT	MUT	WT	SNP6.0
DIPG10	7.6	M	0.23	GBM	Pre	–	K27M	–	–	–	250K
DIPG13	6.6	F	0.83	GBM	Post	WT	K27M	WT	MUT	WT	SNP6.0
DIPG14	5.2	M	1.57	AA	Post	–	K27M	–	–	–	–
DIPG15	11.3	M	0.26	GBM	Post	–	K27M	WT^b^	–	–	–
DIPG17	6.4	F	0.46	GBM	Post	WT	K27M	WT	MUT	WT	SNP6.0
DIPG18	8.0	F	0.03	GBM	Post	–	K27M	–	–	–	–
DIPG20	6.4	M	0.92	GBM	Pre	–	K27M	WT^b^	–	–	–
DIPG21	2.4	F	1.43	AA	Post	WT	K27M	WT	WT	WT	SNP6.0
DIPG22	3.9	F	0.59	LGA	Post	WT	K27M	WT	WT	WT	SNP6.0
DIPG23	6.6	M	0.78	GBM	Post	WT	K27M	WT	MUT	WT	SNP6.0
DIPG24	7.8	M	0.37	AA	Post	WT	K27M	–	MUT	WT	SNP6.0
DIPG26	3.9	M	0.09	GBM	Post	WT	K27M	–	WT	WT	SNP6.0
DIPG27	4.4	F	0.19	LGA	Post	WT	K27M	–	–	–	–
DIPG29	8.1	M	1.73	GBM	Post	WT	K27M	–	MUT	WT	SNP6.0
DIPG30	7.6	F	1.06	GBM	Post	WT	K27M	–	MUT	WT	SNP6.0
DIPG31	14.2	F	1.60	GBM	Post	WT	K27M	–	–	–	SNP6.0
DIPG32	14.0	F	0.52	GBM	Post	–	K27M	–	–	–	–
DIPG34	8.0	M	0.83	GBM	Pre	–	K27M	–	–	–	–
DIPG35	11.0	F	1.17	GBM	Pre	WT	K27M	–	–	–	–
DIPG36	6.0	F	–	GBM	Pre	–	K27M	–	WT	WT	–
DIPG37	13.0	F	1.08	GBM	Pre	–	K27M	MUT	MUT	WT	–
DIPG39	17.0	M	0.58	AA	Pre	–	K27M	–	–	WT	–
DIPG40	4.0	M	0.08	GBM	Pre	–	K27M	WT	–	WT	–
DIPG41	16.3	F	–	GBM	Pre	–	K27M	–	MUT	WT	–
DIPG42	7.0	M	–	GBM	Pre	–	K27M	–	MUT	WT	–
DIPG01	5.0	F	0.42	PNET	Post	WT	WT	WT	MUT	WT	SNP6.0
DIPG02	0.0	F	0.03	PNET	Pre	WT	WT	WT	–	–	250K
DIPG03	4.6	M	1.48	GBM	Post	WT	WT	–	–	–	SNP6.0
DIPG09	6.5	M	4.11	GBM	Post	WT	WT	–	MUT	WT	SNP6.0
DIPG11	1.7	M	6.28^a^	GBM	Pre	WT	WT	WT^b^	–	–	250K
DIPG12	0.3	F	1.68	AA	Pre	WT	WT	WT^b^	–	–	–
DIPG16	5.8	M	0.31	GBM	Pre	WT	WT	WT	MUT	WT	SNP6.0
DIPG19	3.1	F	0.21	AA	Post	WT	WT	–	–	–	–
DIPG25	7.1	F	1.43	AA	Post	WT	WT	WT	WT	WT	SNP6.0
DIPG28	15.2	F	15.88^a^	AA	Pre	WT	WT	WT^b^	–	–	–
DIPG33	2.5	M	13.23^a^	AA	Pre	WT	WT	WT^b^	–	–	–
DIPG38	3.0	M	10^a^	GBM	Pre	WT	WT	WT	–	–	–

*Age Dx* age of diagnosis in years, *OS* overall survival in years, *GBM* glioblastoma multiforme, *AA* anaplastic astrocytoma, *LGA* low grade astrocytoma, *K27M* lysine to methionine at residue 27 of H3.3, *WT* wild-type

^a^Patient still alive at last follow-up

^b^ATRX mutation tested by immunohistochemistry

### Sanger sequencing

Coding exons of *H3F3A*, *HIST1H3B*, *ATRX*, *TP53* and *IDH1* and *2* were sequenced using Sanger fluorescent sequencing after amplification by polymerase chain reaction using standard methods, at The Hospital for Sick Children or McGill University/Genome Quebec Centre (primer sequences in Supplementary Table 1). The *TP53* gene was sequenced for the entire coding sequence (exons 2–11) and the spanning intron–exon junctions with primers as previously described [24]. Sequences were analyzed using Applied Biosystems’ 3730xl DNA Analyzer technology.

### Array hybridization and data analysis

Twenty samples were hybridized to the Genome-Wide Human SNP Array 6.0 and three to the Human Mapping 250 SNP Nsp Array from Affymetrix (Santa Clara, CA, USA) (Table 1). The sample preparation, including DNA extraction, digestion, labelling and hybridization, was performed as directed by the manufacturer. Data were analyzed using Partek Genomics Suite v6.4 (Partek Incorporated, St. Louis, MO, USA) and Genotyping Console 4.1 (Affymetrix), GISTIC2.0 (Broad Institute, Cambridge, MA, USA).

### Immunohistochemistry

Formalin-fixed paraffin-embedded (FFPE) sections were immunohistochemically stained for nuclear ATRX as previously described [23]. 5-μm sections were cut from paraffin blocks and mounted on positively charged microscope slides. Following an overnight incubation at 60 °C, the slides were de-waxed in xylene and hydrated by washes in decreased concentration of ethanol in distilled water. Sections were heat treated in 10 mM citrate buffer for the purpose of antigen retrieval and blocked for endogenous biotin and peroxidase. The tissue sections were incubated at 4 °C overnight with rabbit anti-human ATRX (HPA 001906; Sigma-Aldrich, St. Louis, MO, USA) at a 1:600 dilution. Immunodetection utilized 3,3′diaminobenzidine (DAB) and counterstaining was conducted with hematoxylin. The sections were scored for nuclear ATRX positivity by two independent observers blinded to the clinical data.

### Statistical analysis

Analyses were performed using GraphPad Prism 5 software (La Jolla, CA, USA). When appropriate, two group comparisons were analyzed with two-sided Fisher’s exact test, and continuous scale data were analyzed with unpaired two-tailed Student’s *t* test. Overall survival curves were analyzed using the Kaplan–Meier method and the log-rank test was used to make univariate assessments of Kaplan–Meier plots. *P* value ≤0.05 was considered significant. Multivariate analysis was done using multivariate Cox proportional hazards models and significance testing (α = 0.05) based on the Wald test. Analysis of significant focal amplifications and deletions was conducted using GISTIC 2.0 (Broad Institute, Cambridge, MA, USA) with significance being assigned to regions with false discovery rate ≤5 % (*q* ≤ 0.05).

## Results

### Histone H3.3 mutations are frequent in DIPG

**Fig. 2:**
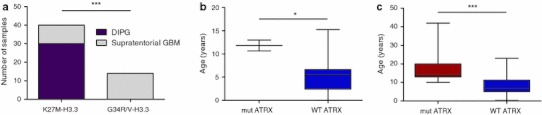
K27M-H3.3 is prevalent in DIPG and is associated with ATRX mutations mainly in older children. **a** Distribution of DIPG and supratentorial GBM based on H3.3 mutations suggests prevalence of K27M-H3.3 in DIPG. *ATRX* mutations in DIPG (**b**) and all location pediatric GBM (**c**) are significantly more prevalent in tumors from older children (mean ages 11.82 (±1.18) years and 16.91 ± 2.11, respectively) as compared to children with no *ATRX* mutation (mean ages of 5.20 (±0.81) years and 8.00 ± 0.69, respectively) (*p* = 0.02 and *p* < 0.0001, respectively)

We sequenced *H3F3A* in 42 DIPG samples comprising either biopsy material prior to any treatment (*n* = 16) or autopsy samples (*n* = 26, one sample from untreated patient at autopsy; DIPG02). We identified the recurrent mutation in Histone H3.3 leading to K27M amino acid substitution in 30/42 (71 %) DIPGs (Table 1). K27M-H3.3 was identified in pre-treatment biopsy samples as well as autopsy material indicating that it is present at diagnosis and not induced by therapy. No *HIST1H3B* (0/29, including 12 of the H3.3 wild-type patients tested for this mutation), *IDH1* (0/23) or *IDH2* (0/20) mutations were identified in our sample set. G34V/R-H3.3 previously identified in 13 % of pediatric and young adult supratentorial GBM was absent in DIPG. The K27M-H3.3 mutation was more prevalent in DIPGs (71 %) compared to supratentorial GBMs (14 %) (Fig. 2a, *p* < 0.0001).

### ATRX mutations are associated with older patient age

We previously showed that G34V/R-H3.3 GBM samples universally also carried *ATRX* and *TP53* mutations (13/13), while K27M-H3.3 GBM samples had significant, albeit lower, overlap with *ATRX* and *TP53* mutations (respectively, 30 and 60 % of the 10 samples investigated) [23]. In the current study, using sequencing and/or immunohistochemical analysis, we identified 2/22 DIPG samples with *ATRX* mutation or loss of immunostaining (suggestive of an underlying mutation) (Table 1). Both cases with ATRX mutation also harbored the K27M-H3.3 mutation. In DIPGs, *ATRX* mutations tended to be found in older children [mean age 11.82 (±1.18) vs. 5.20 (±0.81) years, *p* = 0.02 for *ATRX* mutant versus wild-type DIPGs; Fig. 2b]. The same age-associated distribution of *ATRX* mutation was identified across the entire GBM cohort including both DIPGs and supratentorial GBM [mean age 16.91 (±2.11) vs. 8.00 (±0.69) years, *p* < 0.0001 for *ATRX* mutant versus wild-type cases; Fig. 2c).

### TP53 mutations are frequent in both H3.3 mutant and wild-type DIPGs

As previously described, a significant number of DIPG samples carried mutations in *TP53*, (17/22, 77 %). Fourteen of these samples carrying *TP53* mutations were also mutant for K27M-H3.3 (Table 1). However, even though there was overlap between K27M-H3.3 and *TP53* mutations in DIPGs, there was no significant difference in the frequency of *TP53* mutations between the K27M-H3.3 mutated and wild-type groups (78 and 75 %, respectively).

### DIPG subgroups based on H3F3A mutation status show differing copy number alterations

**Fig. 3:**
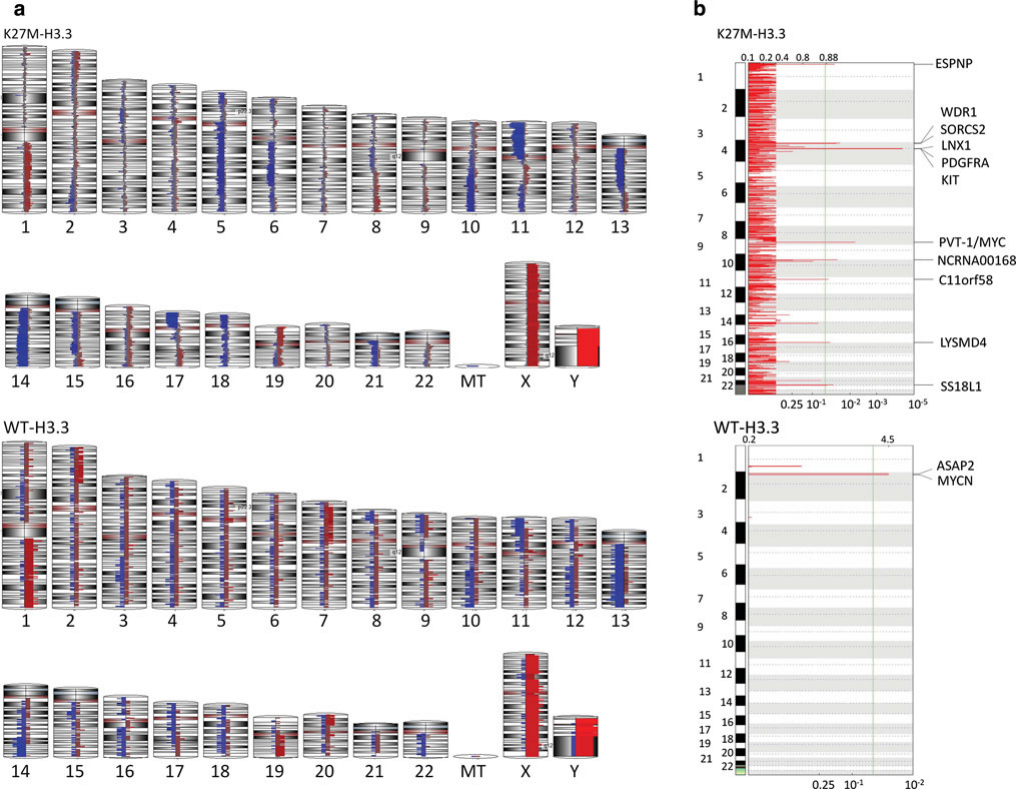
Whole chromosome view of copy number alterations (CNA) in K27M-H3.3 mutants and wild-type DIPG samples. **a** Similarities in CNA between both groups included loss of 10q, 11p, 13q and 14q as well as gains of 1q and 19q. However, major differences in copy number were identified with samples wild-type for K27M-H3.3 exhibiting gains of chromosome 2p and 7p as well as losses of chromosome 9p and 12q while samples mutants for K27M-H3.3 commonly exhibited loss of chromosome 5q, 6q, 17p and 21q. **b** Focal recurrent amplifications determined by GISTIC 2.0 analysis (*q* ≤ 0.05) show significant differences in focal gains between samples carrying K27M-H3.3 and samples wild type for H3.3. This included *PDGFRA* (4q12), *MYV/PVT1* locus (8q24.21) gains and amplifications, which were exclusively identified in K27M-H3.3 mutants and *ASAP2* (2p25.1) and *MYCN* (2p24.3) gains and amplifications which were exclusively identified in wild-type patients

Analysis of DNA copy number alterations in K27M-H3.3 versus H3.3 wild-type DIPG samples showed not only the areas of overlap but also major differences between both groups. Large chromosomal copy number alterations common to both groups included loss of 10q, 11p, 13q and 14q as well as gains of 1q and 19q (Fig. 3a). H3.3 wild-type tumors showed large scale gains of chromosomes 2p and 7p as well as losses of chromosome 9p and 12q. Samples carrying the K27M-H3.3 mutation exhibited common loss of chromosome 5q, 6q, 17p and 21q (Fig. 3a). Gains in the mutated group included 19p.

Focal recurrent gains and deletion in both groups were further analyzed using GISTIC2.0. H3.3 wild-type patients had significant focal gains/amplifications of regions 2p25.1 (*q* = 0.028) and 2p24.3 (*q* = 0.028) including the *ASAP2* and *MYCN* genes, respectively (Fig. 3b). No significant recurrent focal copy number losses were observed in this group (Supplemental Fig. 1). Analysis of frequent focal copy number alterations in the K27M-H3.3 group revealed amplification of 4q12 (*q* = 0.00015) and 8q24.21 (*q* = 0.033) corresponding to *PDGFRA* and *MYC/PVT1* locus gains/amplifications, respectively (Fig. 3b). Interestingly, *PDGFRA* gains/amplifications were found in 40 % (6/15; 1 pre-treatment, 5 post-treatment) of patients in the H3.3 mutant group, while no gains/amplifications of *PDGFRA* were detected in the H3.3 wild-type group. Significant areas of focal deletion in K27M-H3.3 samples included 4p16.3 (*q* = 0.021), 11p15.4 (*q* = 0.000028), 11q22.1 (*q* = 0.022) and 15q24.1 (*q* = 0.016) (Sup. Fig. 1).

### Histone H3.3 wild-type status is associated with better overall survival

H3.3 mutational status and survival data were available for 39 DIPG patients, 27 of whom (69 %) carried the K27M-H3.3 mutation. The mean overall survival for patients with K27M-H3.3 mutated tumors was 0.73 years (±0.48) versus 4.59 years (±5.55) (*p* = 0.0008) for patients with wild-type tumors. Kaplan–Meier survival analysis revealed significantly worse overall survival of DIPG patients carrying the K27M-H3.3 mutation (Log-rank *p* = 0.0027 vs. wild-type patients) (Fig. 1b). Similarly, when patients were stratified by underlying histologic diagnosis (anaplastic astrocytoma versus GBM), Kaplan–Meier survival analysis also demonstrated significantly worse overall survival of DIPG patients carrying the K27M-H3.3 mutation (Log-rank *p* = 0.013 vs. wild-type patients). All of the long-term survivors were included in the H3.3 wild-type group. The mean age of diagnosis of patients with K27M-H3.3 mutations was 8.13 (±3.75) years versus 4.57 (±4.07) years for the wild-type patients (*p* = 0.010). The distribution of age of diagnosis based on K27M-H3.3 mutational status is shown in Fig. 1c. Multivariate analysis (Cox regression), including age, histologic diagnosis and H3.3 mutation status, demonstrated H3.3 mutation status to be the only significant predictor of overall survival with a hazard ratio of 4.3 (95 % confidence intervals 1.3–14.5, *p* = 0.019) (Table 2).

**Table 2:** Multivariate Cox regression analysis

Variable	HR	*p* value	Lower 95 % CI	Upper 95 % CI
K27-H3.3	4.277	0.019	1.264	14.472
Histology	1.93	0.246	0.636	5.862
Age Dx	0.96	0.608	0.823	1.121

*Age Dx* age of diagnosis in years, *HR* hazard ratio, *CI* confidence interval

## Discussion

Our findings confirm that the K27M mutation in histone H3.3 is a frequent event in pediatric DIPG. We further show that both the type of H3.3 mutation and their association with *ATRX* mutations are age and location dependent. The G34V/R-H3.3 mutation was not found in DIPGs whereas it represents 13 % of H3.3 mutations in supratentorial GBM. A recent report similarly did not find mutations at the G34 residue of H3.3 in DIPG [25]. Similarly, *ATRX* mutations were infrequent in DIPG but were present in 29 % of supratentorial GBM. This may be an age-related phenomenon as the mean age of our DIPG cohort was 7.1 versus 12.24 years for the supratentorial GBM patient cohort. In support of this, G34V/R-H3.3 was seen in older patients [mean age 19.66 years (±1.56)] and almost exclusively in hemispheric GBM [23]. Similarly, *ATRX* mutation-positive patients were significantly older than wild-type patients. We did not identify K27M-H3.1, which has been recently identified in 18 % (9 samples) of DIPGs [25]. This difference in frequency may be due to sampling bias; however, our findings support H3.3 as the major histone to be targeted in pediatric GBM and K27 the major residue affected in DIPG.

H3.3 is the major histone to be loaded on chromatin during brain development. This histone variant is known to modulate specific chromatin changes and gene expression profiles and to be associated with active chromatin and translation. Histone lysine methylation has emerged as an important player in regulating gene expression and chromatin function [14]. K27 is a critical residue in all seven histone 3 variants and the subject of post-translational histone modifications as it can be both methylated and acetylated [2, 14, 20]. Acetylation may induce active transcription, while mono, bi or tri-methylation of K27 is associated with a repressive mark on chromatin and gene expression. Abrogation of acetylation and/or potential mimicry of a methylated lysine through the methionine substitution are likely to interfere with chromatin function, inducing defects in chromatin remodelling and tumorigenesis. This is supported by our observation of specific copy number changes associated with mutant K27M-H3.3. Further studies aiming to model this mutation are required to precisely determine the effect of this mutation in chromatin remodelling in pediatric GBM.

*ATRX* mutations were only identified in 9 % of DIPGs compared to 29 % of supratentorial pediatric GBM. Notably, the presence of *ATRX* mutation significantly overlapped with *TP53* mutations in GBM (*p* = 0.01) regardless of the location within the brain and with G34V/R mutants in supratentorial GBM (*p* < 0.0001), and were age-dependent as they mainly occurred in older children (*p* < 0.0001). The requirement for *ATRX* mutations in GBM may thus be due to tumor location and/or the age of the patient. This is potentially indicative of a different cell of origin or age-related plasticity of the tumor, similar to differences in genetic alterations seen based on age in infant MLL-positive leukemia [5]. *TP53* mutations are associated with the vast majority of both K27M and G34V/R H3.3 mutations identified in pediatric and young adult GBM. In both DIPG and supratentorial GBM, *TP53* alterations were commonly identified (77 and 54 %, respectively). Interestingly, in DIPGs, the K27M-H3.3 and wild-type H3.3 subsets had similarly high *TP53* mutation and allelic loss rates. In K27M-H3.3-mutated tumors, this may thus represent an important second hit; however, our data are also indicative of an important role of *TP53* mutations in the pathogenesis of GBM independent of H3.3 mutational status.

One of the most common copy number gains reported in multiple genomic studies of DIPG and pediatric GBM is that of *PDGFRA* [17, 18, 26]. Here, we report *PDGFRA* gain or amplification to be seen exclusively in the patients carrying K27M-H3.3 mutations, where it is present in 40 % of cases. We also identify gains and amplifications in a gene locus containing *MYC/PVT1*, also exclusively in K27M-H3.3 mutants. PVT-1 is an oncogene and a Myc protein target known to be over-expressed in transformed cells [6]. Amplification of *MYC/PVT1* has been shown to contribute to the pathogenesis of ovarian and breast cancer, and is part of the chromosome 8q24 prostate cancer risk locus [16]. The finding of these copy number changes in a subset of K27M-H3.3 mutants suggests that *PDGFRA* and *MYC/PVT1* locus gains/amplifications are subsequent to K27M-H3.3 mutations. The addition of histone modifying agents to RTK inhibitors may thus be of therapeutic benefit in this group of patients.

A clinically significant finding of this study is the fact that patients who harbor the K27M-H3.3 mutation have worse overall survival when compared to patients who are wild-type for H3.3. This association with survival was independent of patient age and histologic diagnosis. The only attributable histologic feature exclusive to the K27M mutant group is glial differentiation. However, not all of the samples mutated for K27M-H3.3 met criteria for GBM. The hypothesis that mutation status identifies distinct subtypes is further supported by the differences in copy number profiles and age distribution between these patient groups. Importantly, long-term survivors were only identified in the group of patients who are wild-type for this gene. Some of these patients had an atypical clinical presentation (longer duration of symptoms or atypical radiology) and thus were biopsied, demonstrating high grade histologic features. Despite high grade histology, this group of H3.3 wild-type patients did not follow the expected clinical course of what can be considered classic for children with DIPG, suggesting a potentially different clinical and molecular entity which should be added to the group of “atypical” DIPG when considering clinical trial design. These H3.3 wild-type tumors may be more heterogeneous in terms of histology and biological features than K27M-H3.3 tumors, and may perhaps, with larger numbers, be further divided into different sub-groups. One uncommon subgroup of H3.3 wild-type brainstem tumors which is hinted at by our series is the PNET. Interestingly, the two PNET patients in our cohort presented with “classical” DIPG features were treated as DIPG patients and both had a poor outcome despite the H3.3 wild-type status. This raises the hypothesis that H3.3 wild-type status may suggest better outcome only in glial neoplasms.

In contrast, the H3.3-mutated group contained patients who, at autopsy, had tumors which, if classified by WHO guidelines, would be considered diffuse astrocytoma, grade II. Nevertheless, these patients had the short survival expected of classic DIPG. Conversely, some patients with high-grade histology such as those with features of GBM were in the wild-type group. Thus, mutational status of H3.3 may be more helpful than histologic appearance alone in identifying patients expected to have a poor clinical outcome at presentation.

Our findings support performing a stereotactic biopsy, particularly for atypical clinical cases of DIPG. The finding of the K27M-H3.3 mutation can be considered as diagnostic of classic DIPG with its expected poor outcome. These patients may benefit from agents targeted at chromatin remodelling and/or histone post-translational modifications with an additional RTK inhibitor. Although not universally indicative of better clinical behavior, wild-type patients should perhaps be considered as atypical DIPG, and if coupled with atypical radiology and/or clinical presentation may warrant a different therapeutic approach.

## Electronic supplementary material

Below is the link to the electronic supplementary material.

Supplementary material 1 (PPTX 134 kb)

Supplementary material 2 (PPTX 281 kb)
