# Supplementary figures and images for: K27M mutation in histone H3.3 defines clinically and biologically distinct subgroups of pediatric diffuse intrinsic pontine gliomas
Source: Acta Neuropathol. 2012 Jun 3;124(3):439–47. doi: 10.1007/s00401-012-0998-0 (PMC3422615; doi:10.1007/s00401-012-0998-0)

## Slide 1
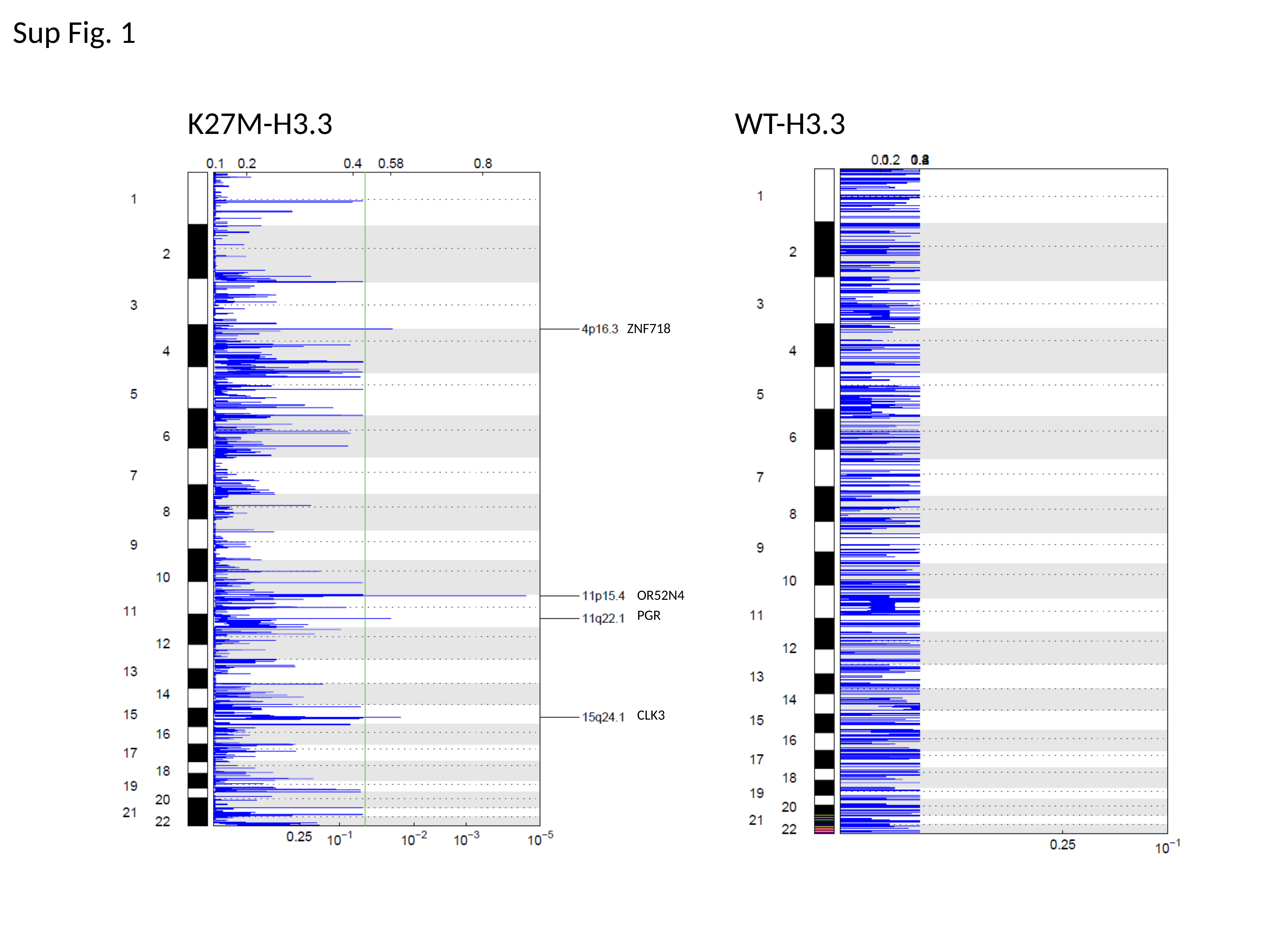

Sup Fig. 1
K27M-H3.3 WT-H3.3
ZNF718
OR52N4
PGR
CLK3

Supplement: Supplementary file 1 — Supplementary material 1 (PPTX 134 kb) [file 401_2012_998_MOESM1_ESM.pptx]

## Slide 1
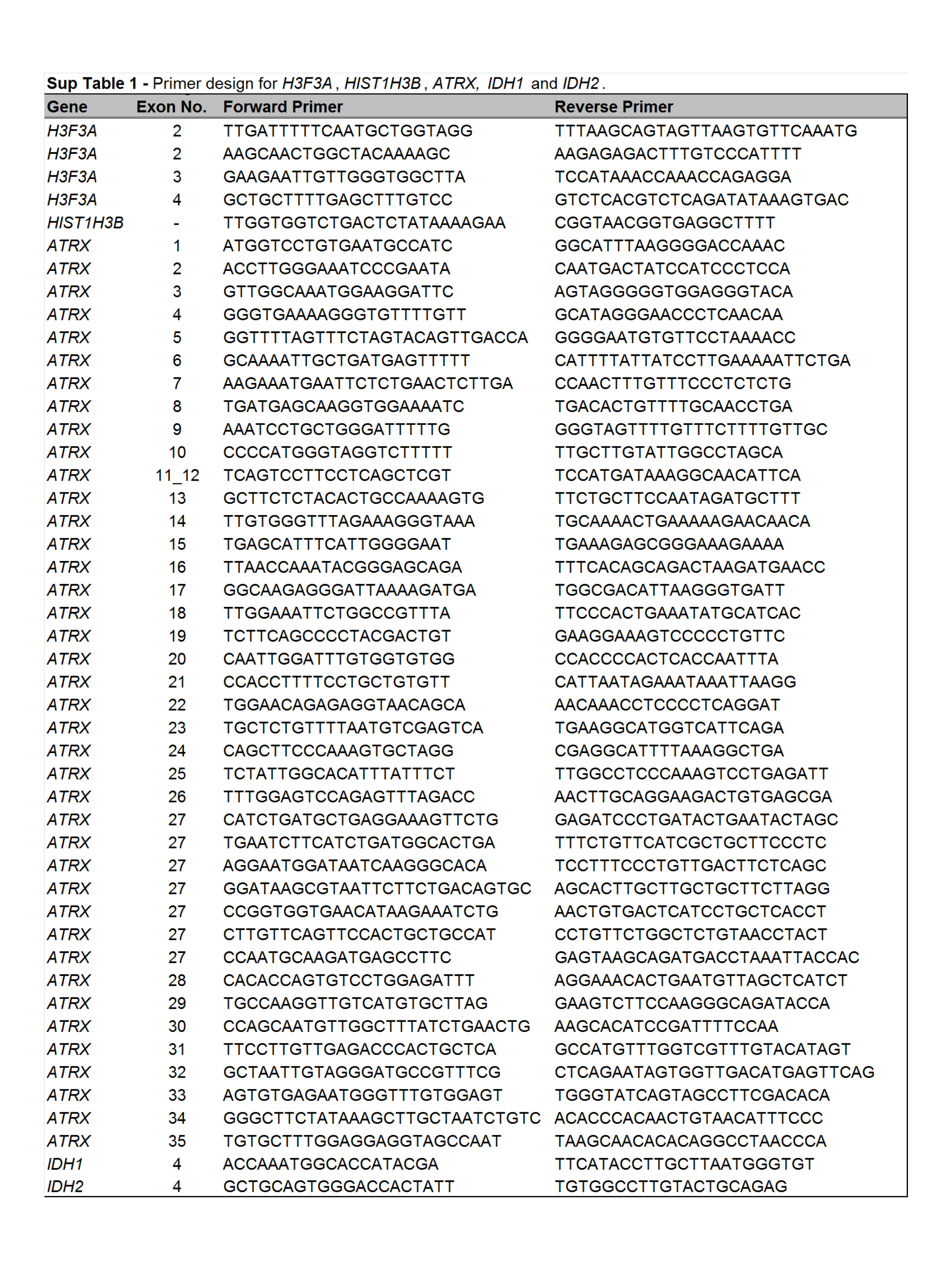

Supplement: Supplementary file 2 — Supplementary material 2 (PPTX 281 kb) [file 401_2012_998_MOESM2_ESM.pptx]
